# Comparison of knowledge, attitudes and practices on exclusive breastfeeding between primiparous and multiparous mothers attending Wajir District hospital, Wajir County, Kenya: a cross-sectional analytical study

**DOI:** 10.1186/s13006-018-0151-3

**Published:** 2018-03-02

**Authors:** Mahat Jimale Mohamed, Sophie Ochola, Victor O. Owino

**Affiliations:** 1 Department of Food, Nutrition and Dietetics, Kenyatta University, P.O BOX 438433, Nairobi, 00100 Kenya; 2 Department of Human Nutrition and Dietetics, Technical University, P.O. BOX 52428, Nairobi, 00200 Kenya

**Keywords:** Knowledge, Attitude and practices (KAP), Primiparous mothers, Multiparous mothers, Exclusive breastfeeding, Kenya

## Abstract

**Background:**

Exclusive breastfeeding (EBF) is recommended for 6 months of age, with continued breastfeeding for 2 years of age or beyond. There is paucity of information on the disparity in Knowledge, Attitudes and Practices (KAP) on EBF between primiparous and multiparous mothers. This study compared the KAP on EBF between primiparous and multiparous mothers attending Wajir County Hospital, Wajir County, Kenya and investigated the association between maternal knowledge and attitudes and EBF.

**Methods:**

Information on maternal KAP on EBF was collected through structured researcher administered questionnaires for a total of 281 mothers, recruited from a maternal and child health centre in 2014; primiparous (*n* = 137) and multiparous (*n* = 144) with infants 0–5 months of age. Maternal knowledge and attitudes on various aspects of breastfeeding were determined. The knowledge and attitude scores were also calculated. The practice of EBF was determined based on a 24-h recall.

**Results:**

The prevalence of EBF among infants 0–5 months old was 45.5%. The rate of EBF among primiparous mothers was 39.4% and multiparous mothers 49.3%. The knowledge score on breastfeeding (out of a total of 10) for the primiparous mothers was 7.93 ± 2.10 and 7.49 ± 2.20 for the multiparous mothers. The mean attitude score (out of a total score of 40) for the primiparous mothers was 29.46 ± 5.65 and 28.65 ± 6.40 for the multiparous mothers. The prevalence of EBF and maternal knowledge and attitudes towards breastfeeding was similar among the two groups of mothers. Those mothers with positive attitudes towards breastfeeding were more likely to EBF (Fisher’s exact test; *p* = 0.00) compared with those with lower scores.

**Conclusions:**

Interventions to promote exclusive breastfeeding should be tailored to the needs of each population by identifying the factors that influence the practice in a given context. The findings of this study will be useful particularly for behavior change communication interventions by those organizations working in similar circumstances to the study area.

## Background

The WHO and UNICEF recommend exclusive breastfeeding (EBF) for the first 6 months of life [1]. Exclusive breastfeeding is defined as giving no other food or drink to the infant, not even water, except breast milk (including milk expressed or from a wet nurse) for the first 6 months of life, but allows the infant to receive ORS (oral replacement solution), drops and syrups (vitamins, minerals and medicines) [2]. The World Health Assembly (WHA) has set a global target to increase the rate of exclusive breastfeeding globally to 50% by 2025 [3]. Exclusive breastfeeding is regarded as one of the most powerful tools policy-makers have at their disposal to improve the health of their population and economies [4].

Globally, only 38% of infants are exclusively breastfed [5]. In East Africa, the EBF rates are quite impressive with Rwanda (84.9%), Burundi (69.3%), Uganda (63.2%), Kenya (61.4%) and Tanzania (50%) all having more than half of the infants 0–5 months old exclusively breastfed [5]. Despite the recent increase in EBF to 61.4% [6] from 32% [7], Kenya has the second lowest rate among the East African countries. Exclusive breastfeeding rates in Wajir County Kenya, the study site is 43.6% which is way below the national rate and the WHO target of 90% [8].

In Kenya, the Ministry of Health targets an annual increase of 3% for the country translating to a rate of 56% by the years 2016/2017 [9]. The Kenya National Nutrition Action Plan 2012–2017 has mainstreamed promotion of exclusive breastfeeding as one of the priority nutrition interventions in the country [9]. Currently, Infant and Young Child Feeding (IYCF) counselling is conducted at government health facilities during growth monitoring and promotion of children less than 5 years of age to mothers and/or caregivers of children already at risk of malnutrition. Counselling on IYCF has also been included in the roles of Community Health Volunteers (CHVs) under the umbrella of the “Community Health Strategy”. Other community based initiatives include the Baby Friendly Community Initiative and Mother-to-Mother Support groups [9].

Primigravidas are regarded as a vulnerable group in which insufficient messages may lead to decreased chances to achieve EBF [10]. Primigravidas are more accepting of non-scientific health promotion messages received through various sources [11]. Primiparous mothers, compared to multiparous mothers, have been observed to have more challenges in practicing EBF being their first experience. Primiparous mothers are less likely to practice exclusive breastfeeding through to 6 months and less likely to breastfeed for 2 years and more [12]. They may have difficulties in adjusting to the new role and less breastfeeding skills [13]. Among the primiparous mothers, factors shown to influence or predict EBF are breastfeeding self-efficacy, breastfeeding outcome expectancy, sociocultural factors and early initiation of breastfeeding [14]. Many studies have demonstrated that multiparity is associated with longer exclusive breastfeeding [15–19]. Studies have shown that breastfeeding duration increases with increasing parity and this may be because of previous breastfeeding experiences [20–22]. In contrast, some studies have shown that parity has no significant influence on duration of breastfeeding [23].

No studies have been conducted in Kenya comparing the Knowledge, Attitudes and Practice (KAP) of EBF among primiparous and multiparous mothers. This study therefore compared the KAP on exclusive breastfeeding between these two groups of mothers and investigated the association between maternal knowledge and attitudes and the practice of EBF.

## Methods

### Study design

A cross-sectional comparative study [24] was adopted for this study with one group composed of primiparous and the second group of multiparous mothers with infants 0–5 months old.

#### Terms used in the study were defined as follows

Prelacteal feed was defined as any food except mother’s breast milk given to a newborn before breastfeeding is initiated [25] and post lacteal feed as any food except mother’s breast milk given to a newborn after the initiation of breastfeeding [25]. Predominant breastfeeding was defined as breastfeeding a child, but also giving small amounts of water or water-based drinks. Neither food-based fluids, nor solid food, nor non-human milk is allowed under this definition) [26].

### Study setting and study participants

The study was carried out at the Maternal and Child Health (MCH) Clinic at Wajir County Hospital, Kenya. Wajir County is in North Eastern Kenya and is mainly inhabited by the Kenyan Somalis who have common cultural practices. The main livelihood activity among the Somalis is pastoralism. Wajir County has a poverty rate of 84.0%, almost double the national rate of 47%. Wajir is ranked the 45th poorest county out of 47 counties in Kenya. Wajir is one of the counties with the highest burden of malnutrition among the under-fives in Kenya with over 26% of the children having stunted growth, 14% wasted and 21% underweight [6].

The study participants were mothers of infants 0–5 months old attending the MCH Clinic at Wajir County Hospital Kenya, during the study period. The exclusion criteria were; mothers with medical conditions or those on medications in which breastfeeding is contraindicated; mothers of infants diagnosed with serious congenital malformations in which breastfeeding was deemed not feasible or contraindicated. These conditions were verified from maternal and child health cards and by the clinicians at the hospital. Mothers were recruited into the study upon informed consent.

### Study variables

The dependent variable in this study was the prevalence of exclusive breastfeeding practice among primiparous and multiparous mothers of infants 0–5 months of age determined by 24-h maternal recall.

The independent variables for the study were maternal knowledge and attitudes towards EBF. Maternal knowledge was tested by 10 questions covering various aspects of breastfeeding. Maternal knowledge score was computed as follows; a correct response was scored one (1) and incorrect responses scored zero (0). The total possible score was 10. Those mothers who scored less than 3 out of 10 were categorized as having poor knowledge, those who scored 3–6 were considered as having moderate knowledge and those who scored 7 and above were categorized as having high knowledge [27].

Maternal attitudes towards EBF was determined by responses to 10 questions. The Likert Scale was used to rate the maternal attitudes and an attitude score calculated. Each correct (positive attitude) response was awarded a score of 4 whereas an incorrect (negative attitude) response got a score of zero (0). To determine the maternal attitudes towards breastfeeding issues, the strongly disagree (SD) and disagree (D) categories were collapsed into one response category (disagree) while strongly agree (SA) and agree (A) into another (agree). The neither agree nor disagree responses were analyzed as a separate category neutral (N). The total possible score for correct responses for all aspects of attitudes tested was 40. The higher the maternal attitude score the more positive the attitude towards breastfeeding. Those mothers who scored less than 20 were categorized as having poor attitudes, those who scored 20–30 had moderate attitudes and those scoring 31–40 as had positive attitudes [27].

### Sample size determination and sampling procedure

The calculated sample size was 280 mothers; 140 primiparous mothers and 140 multiparous mothers. The sample size was calculated using the formula by Cochran [28, 29]. The proportion of the target population estimated to be exclusively breastfeeding nationally was 32% based on the most recent national rate at the time of the study [7].

The sample size was calculated using the formula by Cochran [30] as follows:

$$ \mathrm{n}=\frac{{\mathrm{Z}}^2\mathrm{pq}}{{\mathrm{e}}^2} $$

Where n = the required sample size, Z = the standard normal deviate at 95% confidence level (1.96), *p* = the estimated proportion of the target population estimated to practice EBF (32%) national rate at the time of the study) [7], q = 1-p; and e = desired level of precision (0.05) and a non-response rate of 10%. This yielded a sample size of 334.

Finite population correction was conducted to produce a sample size proportional to the population (a population size less than 10,0000) using the following formula:

$$ \mathrm{n}=\frac{\mathrm{no}}{1+\frac{\left(\mathrm{no}-1\right)}{\mathrm{N}}} $$

Where n = the sample size; no = desired sample size and N = the estimate of the population size (10,000).

This yielded a sample size of 250 (125 per group) which was inflated by a non-response rate of 10% to yield a sample size of 140.

### Sampling techniques and procedure

A systematic random sampling procedure was used for the study. The average number of mothers with infants 0–5 months of age that visited the MCH clinic daily at Wajir district hospital was thirty (30), as reported by the health staff at the MCH clinic. The sampling interval was determined by dividing the average number of mothers (30) that visited the clinic daily with the ideal number of mothers that could be interviewed per day. During the pretest, it was established that the research team (composed of four persons; 3 enumerators and the researcher) could conduct a maximum of 12 interviews per day. The sampling interval was therefore calculated by dividing 30 by 12 to get a sampling interval of 2. On a daily basis, the first mother to be interviewed was selected by simple random sampling technique using a Table of Random Numbers to select a number between 1 and the sampling interval of 2. The randomly selected number (either 1 or 2) represented the first mother to be sampled. The next respondent was selected by adding the sampling interval to the number selected. This procedure was used to select the rest of the mothers to be interviewed during that day. The mothers were sampled from the queue as they waited for health services. The same procedure was conducted on the subsequent days until the required sample size was attained, over a 5-week period. The same sampling procedure was used to select primiparous and multiparous mothers. If the selected mother was not eligible for the study, then the next mother on the queue was selected and the sampling interval maintained.

### Data collection tools

A researcher-administered structured questionnaire with both closed and open ended questions were used to collect data. The questionnaire solicited data on: maternal demographic and socioeconomic characteristics; maternal knowledge, attitudes and practices on breastfeeding; sources of breastfeeding information; and infant feeding practices. Information on breastfeeding practices included; timing of initiation of breastfeeding, feeding on colostrum, giving of prelacteal and post lacteal feeds and exclusive breastfeeding.

The questions were adopted from a face-content validated questionnaire used in Kenya [31] and customized for this study. The questionnaire was developed in English and translated into Somali the local language and then back translated to English to check for consistently and accuracy. The final draft of the Somali language questionnaire was used for data collection.

### Pretesting of data collection tools

The questionnaire was pretested for clarity, validity and reliability at Wajir County Hospital on a sample size of 28 participants (slightly over 10% of the study sample size) after which the necessary adjustments and modifications were made to the tool before being used for the actual study. The participants for the pretest did not participate in the actual study. For reliability, the questionnaire was administered to six mothers who met the inclusion criteria and readministered to the same mothers after a week to determine the consistency of the responses. The calculated reliability coefficient was 0.85. This was within the acceptable range of ≥0.7 (*p* < 0.05) [30]. Content validity was assessed by nutrition experts.

### Data collection procedure and techniques

The researcher and research assistants administered the questionnaire to the sampled mothers in a one-time face to face interview with each study participant at the MCH clinic at the hospital. The interviews were conducted in a private and quiet room at the MCH, Wajir County Hospital. The data collection took place from June to July 2014.

Informed written or thumb print consent was sought from the respondents who were selected to take part in the study. For mothers who were less than 18 years of age assent forms were signed by the parents or guardians before being recruited into the study. Participants were assured of confidentiality.

### Data quality control

Three enumerators (data collectors) with a minimum of a Diploma in Health or Nutrition qualification were recruited from those residing in the study area, and who speak Somali (the local language) and Kiswahili (the national language) fluently. The enumerators also had previous experience in survey data collection. The enumerators underwent a 2 day training to cover the following: explanation of the study objectives, interview techniques, recording of responses and research ethics.

### Data analysis and presentation

The data was checked, cleaned and coded and analyzed using Statistical Package for Social Sciences (SPSS) version 17.0. Descriptive statistics (frequencies, percentages, means and standard deviation) were used to describe maternal demographic and socioeconomic characteristics, prevalence of EBF, knowledge, attitudes and practice of EBF. A t-test was used to establish for significant differences if any, between the primiparous and multiparous mothers for continuous data. Fisher’s exact test was used to test for association between the practice of EBF and categorical variables. A *p* - value of < 0.05 was used as the criterion for statistical significance.

## Results

### Infant feeding practices

#### Demographic characteristics of mothers and children

There was no difference in the age of infants of the primiparous and multiparous mothers (Table 1). The majority of the mothers in the two groups were married. The level of education was low with 43.1% of the mothers having no formal education (Table 1).

**Table 1:** Infant and maternal sociodemographic characteristics

	Primiparous		Multiparous		Total		Chi-square test/t-test
	*n* = 137		*n* = 144		*n* = 281		
	*n*	%	*n*	%	*n*	%	*p*- value
Sex of children:							
Male	74	54	63	43.8	137	48.8	0.11
Female	63	46	81	56.3	144	51.2	
Age of infant in months (mean ±):	2.8 (±1.4)		2.6 (±1.4)		2.7 (±1.4)		0.23
Maternal age in years: (mean ±):	25.1 (±5.3)		27.4 (±5.3)		26.2 (±5.4)		0.00
Marital Status:							
Married	116	84.7	118	81.9	234	83.3	0.08
Single	8	5.8	6	4.2	14	5.0	
Divorced	5	3.7	5	3.5	10	3.6	
Separated	4	2.9	13	9.0	17	6.0	
Widow	4	2.9	2	1.4	6	2.1	
Education:							
No formal education	57	41.6	64	44.4	121	43.1	0.50
Adult education	8	5.8	9	6.3	17	6.1	
Primary education	27	19.7	32	22.2	59	21.0	
Secondary school	14	10.2	15	10.4	29	10.3	
Certificate level	2	1.6	5	3.5	7	2.5	
Diploma level	21	15.3	15	10.4	36	12.8	
Degree level	8	5.8	4	2.0	12	4.3	

### Early infant feeding practices

The majority of the primiparous and multiparous mothers initiated breastfeeding within the first hour after delivery. A higher percentage (53.8%) of multiparous mothers gave prelacteal feeds to their infants compared to primiparous mothers (46.2%). There were no differences in the early infant feeding practices between primiparous and multiparous mothers (Table 2).

**Table 2:** Early infant feeding practices

	Primiparous		Multiparious		Total		Chi-square
	*n* = 137		*n* = 144		*n* = 281		
	*n*	%	*n*	%	*n*	%	*p* - value
Breastfeeding initiation:							
Within 1 h	97	74.1	103	71.5	200	74.1	
> 1 h to < 24 h	32	24.4	32	22.2	64	23.7	
24 h and more	2	1.5	4	2.8	6	2.2	0.44
Gave colostrum 1st 3 days:							
Yes	96	73.3	108	77.1	204	75.3	
No	35	26.7	32	22.9	67	24.7	0.76
Gave prelacteal feeds:	49	46.2	57	53.8	106	37.7	0.23
Prelacteal feeds given:							
Plain boiled water	34	69.4	41	71.9	75	70.8	
Glucose water	10	20.4	12	21.1	22	20.8	
Formula milk	5	10.2	4	7.0	9	8.4	0.85
Reason for giving prelacteal feeds:							
Delayed milk production	42	85.7	46	80.7	88	83.0	
Baby unwell	6	12.3	9	15.8	15	14.2	
Other reasons	1	2.0	2	3.5	3	2.8	0.54
Gave post lacteal feeds:							
Yes	41	30.0	35	24.3	76	27.0	
No	96	70.0	109	75.7	205	73.0	0.44
Post lacteal feeds given:							
Plain boiled water	18	44.0	13	37.1	31	40.8	
Glucose water	9	22.0	5	14.3	14	18.3	
Non-maternal milk	8	19.5	5	14.3	13	17.1	
Formula	2	4.8	3	8.6	5	6.6	
Tea/Juice	3	7.3	7	20.0	10	13.2	
Mashed Vegetable	1	2.4	2	5.7	3	4.0	0.97
Reasons for giving post lacteal feeds:							
Sooth stomachache	7	17.1	11	31.4	18	23.7	
Baby gets hungry	11	26.9	8	22.9	19	25.0	
Advised by relatives	6	14.6	4	11.4	10	13.2	
Mother not producing enough milk	5	12.2	8	22.9	13	17.1	
Advised by healthcare providers	1	2.4	0	0	1	1.3	
Advised by TBA	9	22.0	1	1.0	10	13.1	
Other reasons	2	4.0	3	8.5	5	6.6	0.54

*TBA* Traditional Birth Attendants

### Breastfeeding practices since birth

**Fig. 1:**
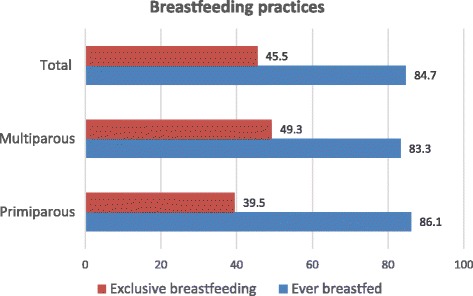
Breastfeeding practices among primiparous and multiparous mothers

The majority of mothers both primiparous and multiparous, reported to have ever breastfed their infants (Fig. 1). Similarly, the majority of the primiparous and multiparous mothers were continuing to breastfeed their infants at the time of the study. Overall, the EBF rate for infants 0–5 months old was 45.5%. The rates of EBF of the primiparous and multiparous mothers were similar, showing no relationship between parity and exclusive breastfeeding rate in this study population.

### Maternal knowledge on exclusive breastfeeding

The majority of the primiparous and multiparous mothers knew that breast milk should be baby’s first feed. Most of the mothers also knew that the baby should be put to the breast within the first hour of birth. The knowledge on breastfeeding among the primiparous and multiparous mothers was similar. The mean knowledge score out of a total of 10 was 7.93 ± 2.10 and 7.49 ± 2.20 for the primiparous and multiparous mothers respectively.

The level of knowledge on various aspects of breastfeeding was high. The lowest level of knowledge was observed on the aspect that touched on whether pregnant mothers should continue breastfeeding their infants or not (Table 3).

**Table 3:** Maternal knowledge on breastfeeding

	Primiparous		Multiparious		Total		Chi-square test
	*n* = 137		*n* = 144		*n* = 281		
	*n*	%	*n*	%	*n*	%	*p -* value
Aspect of Knowledge:							
Breast milk should be baby’s first feed	131	95.6	125	86.8	256	91.1	0.01
Baby should be put to breast within 1 h of birth	127	92.7	130	90.3	257	91.5	0.47
Colostrum should be fed to the baby	120	87.6	122	84.7	242	861	0.49
Breast milk alone can sustain baby for 6 months	107	78.1	113	78.5	220	78.3	0.94
Breast feeding protects baby from illness	112	82.4	114	79.2	226	80.7	0.50
Breast feeding protects mother from pregnancy	99	72.8	100	69.4	199	71.1	0.54
Expressed breast milk should be fed to the baby	99	72.8	96	66.7	195	69.6	0.27
Semi-solid food to be introduced at 6 months	102	75.0	97	67.4	199	71.1	0.16
A pregnant woman can breastfeed her baby	78	57.8	81	56.5	159	54.2	0.85
A baby should be breast fed on demand	104	75.9	102	70.2	206	73.3	0.32

### Maternal attitudes towards exclusive breastfeeding

The majority of the mothers believed that EBF is beneficial to the child, with no difference between the primiparous and multiparous mothers. Similarly, a good proportion of the mothers believed that breastfed babies are healthier than non-breastfed babies and also that breastmilk is more easily digested than animal milk (Table 4). In contrast, negative maternal attitudes towards breastfeeding were also observed among the mothers. A relatively high percentage of mothers believed that infant formula is a better choice for working mothers, implying a negative attitude towards these aspects of breastfeeding (Table 4). There were no differences in maternal attitudes on the various aspects of breastfeeding between the primiparous and multiparous mothers.

**Table 4:** Maternal attitudes towards breastfeeding

Primiparous (*n* = 137)	Multiparous (*n* = 144)						*χ*^2^ (*p* - value)						
Aspects of Attitude	Agree		Disagree		Neutral		Agree		Disagree		Neutral		*p -* value
	*n*	%	*n*	%	*n*	%	*n*	%	*n*	%	*n*	%	
Believe that EBF is beneficial to the child	130	94.9	6	4.4	1	0.7	137	95.1	5	3.5	2	1.4	0.46
The age of the mother influences her ability to EBF	107	78.1	20	14.6	10	7.3	104	72.2	21	14.6	19	13.2	0.28
A baby can survive without water	89	65.0	41	29.9	7	5.1	92	63.9	42	29.2	10	6.9	0.52
Husbands should be involved in decision making on whether to EBF	68	49.6	56	40.9	13	9.5	75	52.1	55	38.2	14	9.7	0.35
Animal milk is suitable for a new born baby	74	54.0	51	37.2	12	8.8	61	42.4	63	43.8	20	13.9	0.06
Breast milk is inadequate for babies 2 months or older	73	53.3	51	37.2	13	9.5	80	55.6	57	39.6	7	4.9	0.52
Formula feeding is better choice for working mothers	38	27.7	87	63.5	12	8.8	56	38.9	77	53.5	11	7.6	0.03
Breastfed babies are healthier than fed babies	120	87.6	12	8.8	5	3.6	114	79.2	19	13.2	11	7.6	0.06
Breast milk is more easily digested than formula	118	86.1	13	9.5	6	4.4	110	76.4	21	14.6	13	9.0	0.04
Infant cannot survive without water besides breast milk	56	40.9	73	53.3	8	5.8	56	38.9	75	52.1	13	9.0	0.50
Number of times a mother has given birth will influence her ability to EBF	12	8.8	81	59.1	7	5.1	17	11.8	76	52.8	4	2.8	0.44

### The association between maternal knowledge and maternal attitudes and the practice of exclusive breastfeeding

For the purpose of testing the association between maternal knowledge and attitudes and the practice of exclusive breastfeeding, all those mothers who practiced EBF were pooled together in analysis irrespective of their parity. Similarly, all the mothers who did not practice EBF were pooled together irrespective of their parity. Maternal knowledge was not associated with the practice of EBF (Table 5). Mothers with a positive attitude towards breastfeeding were more likely to exclusively breastfeed their babies than those with negative attitudes (*p* = 0.00) as shown in Table 5.

**Table 5:** The association between maternal knowledge and attitude on breastfeeding with exclusive breastfeeding

	EBF		Fisher’s exact test (*p -* value)
	Yes	No	
	*n* = 125	*n* = 156	
	*n* (%)	*n* (%)	
Knowledge score (out of 10):			
Poor ≤3)	3 (2.4)	8 (5.1)	
Medium (4–6)	30 (24.0)	42 (26.9)	
High (> 7)	90 (73.6)	106 (67.9)	0.42
Attitude score (out of 40):			
Poor ≤20	6 (4.8)	13 (8.3)	
Moderate 21–30	46 (36.8)	96 (61.5)	
Positive > 31	73 (58.4)	47 (30.1)	0.00

## Discussion

Several studies have investigated parity as a predictor of the practice of exclusive breastfeeding [16–21] but to the best of our knowledge our study was the first study in Kenya to compare maternal KAP on EBF between primiparous and multiparous mothers.

In our study, exclusive breastfeeding was measured in the previous 24 h and therefore the true EBF rate since birth would be lower. The study also found no association between EBF and parity.

There is scarcity of information on studies conducted to establish the prevalence of EBF in different areas of Kenya. The findings on the EBF rate in our study are similar to that of a study conducted in the same study area (Wajir County) in 2014 by Islamic Relief Kenya [8] using the same definition of EBF as used in our study. Some studies conducted in Kenya, using the same definition of EBF, have however, reported lower rates than reported in our study. A study conducted in Nyando district Kisumu, Kenya reported an EBF rate of 33% [32]. In another study conducted in the informal settlement in Nairobi, an EBF rate of 2% was observed [33]. The findings of an intervention study conducted in an informal settlement in Nairobi reported an EBF rate of only 15.6% after the provision of seven intensive home based counseling sessions [34]. The relatively high rate of EBF reported in our study could be attributed partly to the large presence of Non-Governmental Organizations (NGOs) in the area that are implementing programmes aimed at improving child survival. The NGOs in Kenya work mainly in the Arid and Semi-Arid Lands (ASAL) areas where there is higher vulnerability for malnutrition among mothers and children, due to, not only food insecurity but also to poor accessibility to health services and infrastructure, such as roads and supply of water. The study area (Wajir County) is situated in an ASAL region. The NGOs working in partnership with the Ministry of Health are implementing services under the Community Health Strategy and thus making health and nutrition services more accessible to the population in such areas. The implementation of health and nutrition services under the Community Health Strategy is strong in areas where the NGOs are supporting the Ministry of Health in provision of services at the community level.

The current Kenya national exclusive breastfeeding rate is 61.4% as reported in the 2014 Kenya Demographic Health Survey [6]. The EBF rate in this survey was not only lower than the national rate but was also below the WHO recommended rate of 90% [4] implying that concerted efforts are needed to improve the practice of EBF.

Overall, the mothers’ knowledge on breastfeeding reported in our study was high. Primiparous mothers exhibited a higher knowledge score compared to the multiparous mothers although the difference was not statistically significant. The high knowledge among the mothers could be partly attributed to some of the government strategies to improve EBF rates, such as the Baby Friendly Hospital Initiative (BFHI), Baby Friendly Community Initiative (BFCI) and Mother-to-Mother Support groups. All are the key Ministry of Health and non-Governmental interventions promoting EBF at the national, health facility and community level. These strategies form the major focus of interventions aimed at improving infant and young child feeding practices as stipulated in the Kenya National Nutrition Action Plan [9]. Studies conducted in other areas in Kenya have also demonstrated high maternal knowledge on breastfeeding particularly on the duration of exclusive breastfeeding [33]. The high level of knowledge on breastfeeding did not however necessarily translate into practice implying that factors other than knowledge influenced the choice of infant feeding in this community. The findings of many studies conducted in sub-Saharan Africa, Kenya included, have shown culture as having a major influence on infant feeding practices [35, 36]. It is therefore possible that cultural beliefs related to infant feeding may have contributed to the choice of infant feeding in the study community. Cultural attitudes, beliefs and norms are important factors in the WHO’s model of the determinants of infant and child feeding behavior [35] as they are known to affect breastfeeding practices. Other researchers have identified detrimental cultural beliefs (insufficient milk and “bad” colostrum) as a hindrance to the practice of exclusive breastfeeding [36].

The findings of our study show that maternal attitude towards exclusive breastfeeding was positive with no significant differences between the primiparous and multiparous mothers. The positive maternal attitude towards EBF can probably be explained by the fact that the Ministry of Health and Non-Governmental Organizations dealing with child health emphasise the improvement of breastfeeding practices through various strategies at both the health facility and community level, as well as through the media. Just like the reasons for the high knowledge among the mothers, it is probable the Ministry of Health Strategy and Interventions in improving IYCF practices and specifically EBF could have led to the positive attitudes among the mothers towards exclusive breastfeeding. In our study, those mothers with positive attitudes towards breastfeeding were more likely to exclusively breastfeed their infants.

One limitation of the study, was the use of the 24 h recall period data collection methodology for establishing EBF rate because it over estimates EBF since birth. Exclusive breastfeeding is defined as giving no other foods at all since birth and not just in the last 24 h. This method overestimates the proportion of exclusively breastfed infants, as some infants who are given other liquids irregularly may not have received them the day before the survey. This is however, the method recommended by WHO and used in the DHS globally and therefore allows for comparison of our findings with that of other studies. Appropriate attention was given to ensure quality control of data by thorough training of the enumerators, and close supervision of all the study activities.

## Conclusions

There were no significant differences in the knowledge, attitudes and practice of exclusive breastfeeding among primiparous and multiparous mothers. The findings of our study have demonstrated that parity did not influence the practice of exclusive breastfeeding of our study population. Interventions to promote exclusive breastfeeding should be tailored to the needs of each population. Positive attitudes towards breastfeeding were associated with exclusive breastfeeding but maternal knowledge was not. Efforts to promote exclusive breastfeeding should, in addition to improving maternal knowledge and attitudes, focus on the identification of factors that influence the practice and how they can be addressed in a participatory manner by all stakeholders in the community. The findings of our study will be useful particularly to those organizations that focus on behavior change communication interventions, to improve exclusive breastfeeding.

## Abbreviations

ANC: Antenatal care
BFCI: Baby Friendly Community Initiative
BFHI: Baby Friendly Hospital Initiative
CHW: Community health workers
CWC: Child welfare clinic
DHS: Demographic health survey
EBF: Exclusive breastfeeding
FGD: Focus group discussion
IYCF: Infant and young child feeding
KAP: Knowledge, attitudes and practice
KDHS: Kenya demographic and health survey
KNBS: Kenya national bureau of statistics
MCH: Maternal child health
TBA: Traditional birth attendants
